# Adolescents and Long-Acting Reversible Contraception: Lessons from Mexico

**DOI:** 10.1007/s10995-016-2013-1

**Published:** 2016-05-05

**Authors:** Biani Saavedra-Avendano, Zafiro Andrade-Romo, Maria I. Rodriguez, Blair G. Darney

**Affiliations:** 1 Center for Health Systems Research/Centro de Investigacion en Sistemas de Salud (CISS), National Institute of Public Health/Instituto Nacional de Salud Publica (INSP), Av. Universidad, No. 655, 62100 Cuernavaca, Morelos Mexico; 2 Department of Obstetrics and Gynecology, Oregon Health and Science University, Portland, OR USA; 3 Department of Reproductive Health and Research, World Health Organization, Geneva, Switzerland

**Keywords:** Adolescents, LARC, Mexico, IUD

## Abstract

*Objectives* We describe current use of long acting reversible contraception LARC (tier 1), hormonal (tier 2), barrier and traditional contraceptive methods (tier 3) by adolescent women in Mexico. We test whether knowledge of contraceptive methods is associated with current use of LARC.* Methods* We used the 1992, 1997, 2006, 2009 and 2014 waves of a nationally representative survey (ENADID). We used information from n = 10,376 (N = 3,635,558) adolescents (15–19 years) who reported ever using any contraceptive method. We used descriptive statistics and logistic regression models to test the association of knowledge of method tiers with use of tier 1 (LARC) versus tier 2, tier 3, and no contraceptive use.* Results* Over time, LARC use in the overall sample was flat (21 % in 1992, 23 % in 2014; *p* = 0.130). Among adolescents who have had a pregnancy, LARC use has increased (24 % in 1992 to 37 % in 2014). Among adolescents who did not report a pregnancy, current LARC use has remained low (1 % in 1992 and 2 % in 2014). We found positive association between LARC use and knowledge of tier 1 methods. In the overall sample LARC use is strongly correlated with exposure to marriage compared to use of tier 2 or tier 3 methods.* Discussion* Among adolescents in Mexico who are currently using modern methods, LARC use is relatively high, but remains primarily tied to having had a pregnancy. Our study highlights the need to expand access to LARC methods outside the post-partum hospital setting.

## Significance

The safety and acceptability of LARC use by all women, including adolescents is well established. Data on LARC use by adolescents, as well as barriers to accessing and accepting a LARC method for adolescents is needed to help guide policy and programmatic care. Our study shows that LARC use in Mexico is relatively high among female adolescents who are currently using modern methods, but remains primarily tied to having had a pregnancy. It also highlights the need to expand access to LARC methods outside the post-partum hospital setting, including education and practical training for health care providers in primary care.

## Introduction

Adolescent pregnancy is a persistent global health problem [9]. Births to adolescents account for 11 % of all births worldwide, and in low- and middle-income countries (LMICs), complications from pregnancy, childbirth, and unsafe abortion are among the leading causes of morbidity and mortality among adolescent females [37]. It is estimated that each year, 71 unintended pregnancies occur per 1000 women of reproductive age in Mexico [26]. Furthermore, while the total fertility rate has fallen drastically, from 6.8 in the 1950s to 2.3 by 2010, likely due to use of modern contraceptive methods [12, 34], adolescent birth rates have stagnated, leading to an increase in the proportion of total births to adolescents. In general, contraceptive prevalence rates among adolescents are low, contributing to the high unintended pregnancy rate among adolescents [38].

Long-acting reversible contraception (LARC), which includes intrauterine devices or systems (IUD/IUS) and implants, is very effective at preventing unintended pregnancy [36]. The safety and acceptability of LARC use by all women, including adolescents is well established [1, 33]. Despite this, LARC use by adolescents remains underutilized globally [17]. Data on current LARC use by adolescents, as well as barriers to accessing and accepting a LARC method for adolescents is needed to help guide policy, programs, and health service delivery.

In Mexico, access to contraception, including for adolescents, is embedded in national policy, which declares that all citizens have the right to family planning [7]. Furthermore, the National Strategy to Prevent Teen Pregnancy states that modern contraceptive methods should be available to adolescents across all types of facilities and insurance payers (employment-based or public) [11]. Policy change alone however, has not been enough to change adolescent birth rates: the proportion of births to women under 20 years of age has actually increased in Mexico over the past decade [20]. This may be in part due to a predominance of permanent contraception in Mexico. Mexican women rely heavily on sterilization, and many choose sterilization once family size has been achieved [2]. Prenatal care is in many cases women’s first encounter with health care system and contraceptive services [16]. However, adolescents and young women seeking to delay or space births need access to effective reversible methods.

An improved understanding of current trends in adolescent utilization of LARC in Mexico can help inform policy and programmatic interventions to prevent unintended pregnancy, both in Mexico and globally. The purpose of this study was to characterize current use of LARC (tier 1), hormonal (tier 2), barrier and traditional contraceptive methods (tier 3) [8, 39] by adolescent females in Mexico over time (1992–2014). We tested the association of knowledge of contraceptive methods by tier and current use of LARC methods (tier 1) compared with use of tier 2, tier 3, or non-use. We hypothesized that knowledge of tier 1 methods would be positively correlated with use of LARC, controlling for socio-demographic and other factors.

## Methods

We used the 1992, 1997, 2006, 2009 and 2014 waves of the *Encuesta Nacional de la Dinámica Demográfica* (ENADID), a cross-sectional population-based survey representative at national and state level (Mexico has 32 states) and rural/urban stratum. The ENADID uses a complex multistage sampling process. Trained interviewers carried out standardized, direct, structured face-to-face interviews with key household informants. All participants gave informed consent [13, 21–24].

**Fig. 1:**
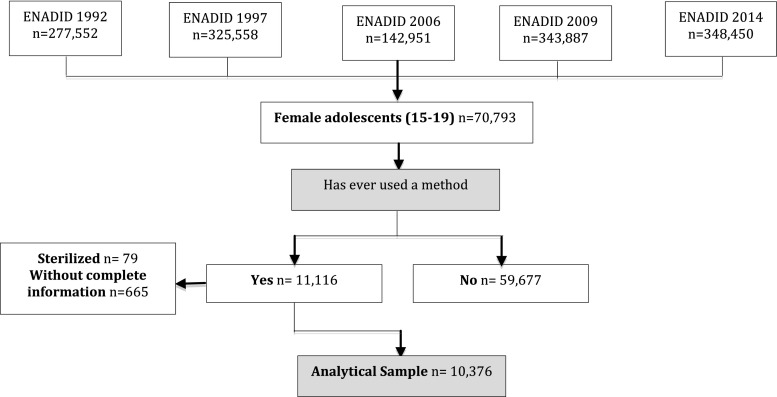
*Top* Definition of analytical sample

Each wave includes survey modules covering household characteristics, composition and demographic, education, and health information on all household members. Each wave also includes a reproductive health module asked of women ages 15–54 residing in the household. We merged the household and individual-level reproductive health modules to extract all study variables. We restricted our sample to non-sterilized female adolescents aged 15–19 years old (Fig. 1).

Our main outcome variable was current use of LARC among women 15–19. Women who reported having ever used any contraceptive method were asked if they currently use a method. More than one response is allowed for the ever used and current use contraceptive questions. We classified current contraceptive method based on the level of effectiveness of the most effective method reported: tier 1 LARC (IUD and implant), tier 2 hormonal methods (pills, injection and patch), tier 3 condom, sponge and traditional methods (withdrawal and rhythm), and no method.

We included several individual and household-level characteristics that may also influence current LARC use. We measured knowledge of contraceptive methods using the tiers classification. Women were asked about their knowledge of contraceptive methods—both spontaneous (open ended question) and with prompting (a list of methods). We classified methods known based on tiers of effectiveness. Most women (78 %) reported knowledge of more than one method in more than one tier. We present descriptive results allowing women to name more than one method. In descriptive analyses, a woman can thus be in more than one tier category and totals sum to >100 %. For our regression models, we created a mutually exclusive categorical variable and classified each woman into the highest tier method she reported knowing. For example, a woman who reported knowing about IUDs and condoms is classified into tier 1, with IUD. We compared knowledge of tier 2 and 3 methods, and no knowledge of any contraceptive method, with knowledge of tier 1 methods.

We measured education by subtracting the number of years of schooling reported by each woman from the number of years of schooling a woman would be expected to have given her age; we call this continuous variable educational gap, and present it in years [25]. We measured ethnicity by whether the woman reported speaking an indigenous language (we do not have an ethnicity variable for the 1992 wave). This is the preferred classification for ethnicity of the Mexican government. We classified women as ever married (including divorced and widowed) or cohabiting versus not. We created an indicator of whether the woman reported employment outside the home in the past week, an indicator of whether the woman reported ever been pregnant, and whether the woman reported having health insurance. Health insurance is classified as employment-based, called *Seguro Social*, public insurance for those working in the informal sector, self-employed, or otherwise without insurance, called *Seguro Popular*, or none. We do not have health insurance information for the 1992 wave.

At the household level, we created an indicator of whether the head of household is male, and the highest level of education completed by the head of household (none, primary, secondary, and high school or more). These are indicators of household socio-economic status, with female-led and lower education-level households having lower socio-economic status. We identified whether the household was in a rural (<2500 inhabitants) location or not, and included an indicator for region based on grouping the 32 Mexican states by average socioeconomic level (Mexico City, is the wealthiest region in México) [27]. Finally, we created an indicator for survey year (1992, 1997, 2006, 2009 and 2014).

Overall, 6 % of the women who reported ever using a contraceptive method did not have complete data. There were no significant differences in patterns of missing data by outcome (data not shown). Most of the missing values were in the 1992 and 1997 waves.

### Analysis

We used descriptive statistics to first examine current use of contraceptive methods by year and pregnancy history among female adolescents. We then classified adolescents as current users of tier 1 (LARC), tier 2 (hormonal methods—pills, injections and patch-), tier 3 (condom, sponge and traditional methods), or non-users. We used Chi square tests to examine differences in all individual and household level variables by outcome (tier 1, 2, 3 or non-users).

We developed three logistic regression models to test the association of knowledge of method tiers with use of tier 1 (LARC) versus tier 2, tier 3, and no contraceptive use, controlling for covariates as described above. Next, we focused on the subsample of adolescents who reported a previous pregnancy, which is a concrete marker for history of sexual activity, and thus potential risk for subsequent unplanned pregnancy. Sixty-seven percent of our analytic sample had experienced a pregnancy. We were unable to run models with the subsample of women who had not experienced a pregnancy due to small cell sizes for categories of contraceptive tiers. Exposure to marriage and previous pregnancy were highly correlated (correlation coefficient = 0.75), and we were therefore unable to include both in a single model.

We performed several sensitivity analyses. We compared results from models using contraceptive knowledge as a dichotomous variable (yes/no), and as a continuous variable (number of methods known); there was no difference in model interpretation. We have neither health insurance nor ethnicity information for the 1992 wave, so we compared models with and without the 1992 data, adding insurance and ethnicity variables to the model. There were no differences in the interpretation of our findings; we present only models without these two variables. We examined interactions between age (15–16 vs. 17–19 years old) and contraceptive knowledge; the interaction was insignificant, suggesting no effect modification of knowledge by age.

We used survey weights to account for the complex survey design in all descriptive and multivariable analyses. We report both analytic and population samples (n and N) and population estimates for all descriptive and multivariable results. We used Stata 13.0 (StataCorp LP, College Station, TX, USA; 2013) for all analyses. This research was conducted in accord with prevailing ethical principles and reviewed by the Institutional Review Board of the National Institute of Public Health in Mexico. This research is not based upon clinical study or patient data.

## Results

**Fig. 2:**
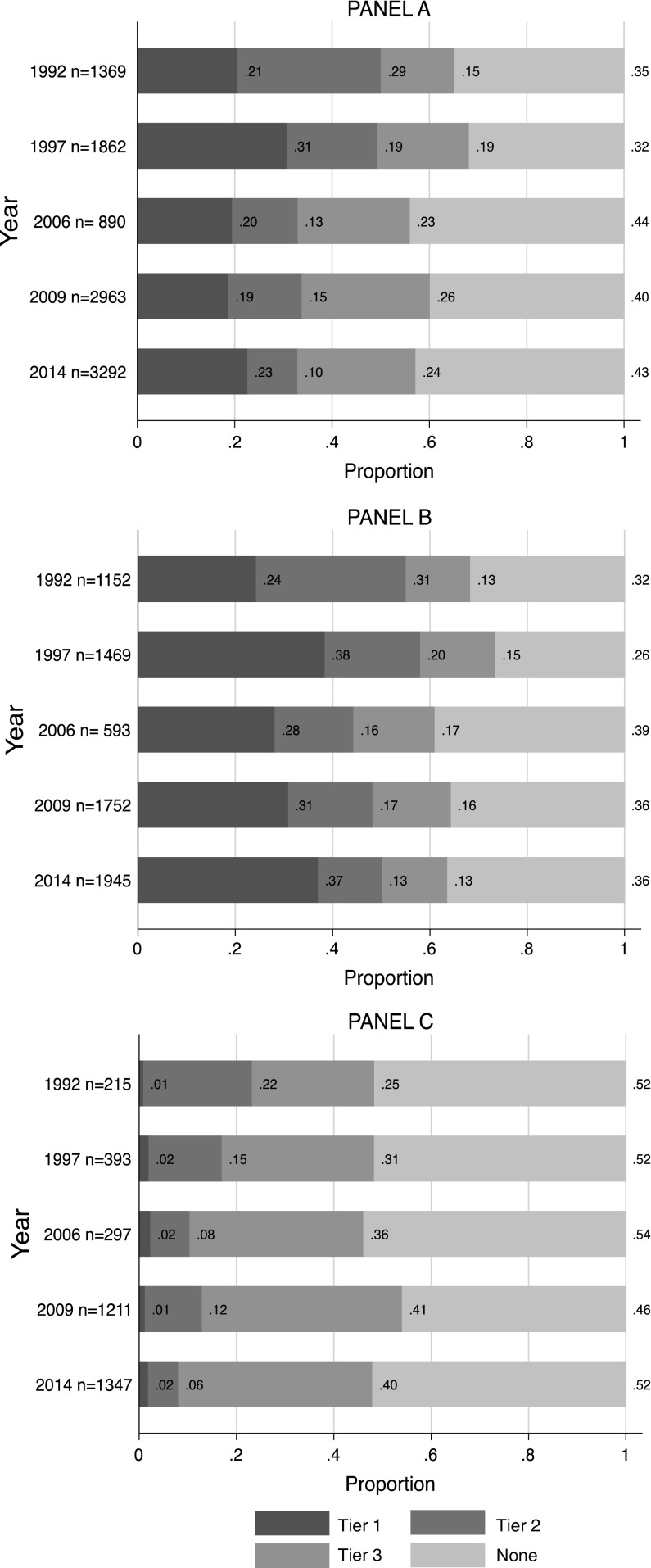
*Top* Proportion of female adolescents who reported current use of contraceptive method by year and pregnancy history (**a** overall sample, **b** ever pregnant, **c** never pregnant), Mexico ENADID 1992–2014. *Note* Among those who report ever using a method

Our analytic sample included 10,376 non-sterilized adolescents (15–19 years old) who reported ever using any contraceptive method (population N = 3,635,558; Fig. 1). Over time, LARC use in the overall sample was flat (21 % in 1992, 23 % in 2014; *p* = 0.130 Fig. 2a). In the subsample of female adolescents who reported a previous pregnancy, current LARC use increased over time (24 % in 1992, 37 % in 2014; *p* = 0.001 Fig. 2b). Among adolescents who had not experienced a pregnancy (Fig. 2c) current LARC use has remained low: 1 % in 1992, 2 % in 2014; *p* = 0.316). Around 50 % of adolescents who have not experienced a pregnancy (52 % in 1992, 52 % in 2014) do not use any contraceptive method, while among those who have experienced a pregnancy, 32 % in 1992 and 36 % in 2014 reported no method. Current use of tier 1 methods has remained overwhelmingly IUD use: implant use has increased over time, but continues to be low (0 % in 1992 to 6 % in 2014; data not shown).

Table 1 shows socio-demographic characteristics of female adolescents who reported ever using any contraceptive method by current tier of contraceptive use; 86 % of our analytic sample was 17–19 years old (Table 1). Among adolescents who reported current LARC use, a higher proportion (90 %) had been exposed to marriage or co-habitation, compared with 85 % of current users of hormonal methods (tier 2), 54 % of current users of condom, sponge or traditional methods (tier 3) and 53 % among non-users (Table 1; *p* < 0.01 for difference by contraceptive method). Results were similar by pregnancy history, which is correlated with marriage; 97 % of current LARC users had experienced a previous pregnancy compared with 77 % of users of hormonal methods (tier 2), 42 % of current users of condom, sponge or traditional methods (tier 3) and 57 % among non-users (*p* < 0.01 for difference by contraceptive use status; Table 1). 74 % of female adolescents who reported ever using any contraceptive method have knowledge of tier 1 methods, 90 % of tier 2 methods, 80 % of tier 3 methods and 3 % do not know any method.

**Table 1:** Socio-demographic characteristics of female adolescents by current contraceptive method use, Mexico ENADID 1992–2014

	Total		Tier 1: LARC		Tier 2: Hormonal (Pills/injection/patch)		Tier 3: Condom/sponge/traditional		None	
n (%)	10,376 (100)		2334 (22.5)		1655 (15.9)		2335(22.5)		4047 (39.0)	
N (%)	3,635,558 (100)		822,363 (22.6)		520,612 (14.3)		852,175 (23.4)		1,439,681 (39.6)	
	*Proportion* (95 % CI)									
Individual characteristics										
Age (17–19)	0.86	(0.85,0.87)	0.88	(0.85,0.90)	0.90	(0.88,0.92)	0.84	(0.81,0.86)	0.85	(0.83,0.86)**
Educational gap^a^ (mean)	1.00	(0.93,1.07)	1.31	(1.19,1.43)	1.32	(1.18,1.46)	0.83	(0.70,0.89)	0.82	(0.73,0.92)**
Married or co-habiting exposure	0.66	(0.64,0.68)	0.90	(0.88,0.92)	0.85	(0.82,0.88)	0.54	(0.51,0.58)	0.53	(0.50,0.55)**
Speaks an indigenous language	0.04	(0.03,0.04)	0.05	(0.04,0.06)	0.04	(0.03,0.05)	0.04	(0.03,0.05)	0.02	(0.02,0.03)**
Currently working	0.21	(0.20,0.23)	0.18	(0.16,0.20)	0.24	(0.21,0.27)	0.22	(0.20,0.25)	0.22	(0.20,0.24)**
Contraceptive knowledge^b,^**										
Tier 1 (Sterilization, LARC)	0.74	(0.73,0.76)	0.90	(0.88,0.92)	0.72	(0.68,0.75)	0.68	(0.65,0.71)	0.70	(0.68,0.72)
Tier 2 (Pills, patch, injection)	0.90	(0.89,0.91)	0.88	(0.86,0.90)	0.94	(0.92,0.96)	0.88	(0.86,0.90)	0.92	(0.90,0.93)
Tier 3 (Condom, sponge, traditional)	0.80	(0.78,0.81)	0.70	(0.67,0.73)	0.69	(0.66,0.73)	0.88	(0.86,0.90)	0.84	(0.82,0.86)
None	0.03	(0.02,0.03)	0.03	(0.02,0.05)	0.03	(0.02,0.05)	0.03	(0.03,0.05)	0.02	(0.02,0.03)
Has ever been pregnant	0.65	(0.64,0.67)	0.97	(0.96,0.98)	0.77	(0.74,0.79)	0.42	(0.39,0.46)	0.57	(0.54,0.59)**
Health insurance**										
None	0.44	(0.42,0.46)	0.40	(0.36,0.43)	0.48	(0.44,0.52)	0.45	(0.42,0.48)	0.44	(0.42,0.47)
Social Security	0.27	(0.26,0.29)	0.24	(0.21,0.27)	0.25	(0.21,0.29)	0.31	(0.28,0.34)	0.28	(0.26,0.30)
Seguro Popular	0.29	(0.27,0.30)	0.36	(0.33,0.40)	0.28	(0.24,0.31)	0.24	(0.21,0.27)	0.28	(0.26,0.30)
Household characteristics										
Man as head of household	0.76	(0.75,0.77)	0.81	(0.79,0.84)	0.80	(0.77,0.83)	0.73	(0.70,0.76)	0.74	(0.72,0.76)**
Education of the head of household										
None	0.06	(0.05,0.06)	0.06	(0.05,0.07)	0.05	(0.04,0.07)	0.04	(0.03,0.06)	0.06	(0.05,0.08)**
Primay	0.41	(0.39,0.43)	0.47	(0.43,0.50)	0.45	(0.41,0.49)	0.38	(0.35,0.41)	0.38	(0.36,0.40)
Secondary	0.31	(0.29,0.32)	0.32	(0.29,0.35)	0.28	(0.25,0.32)	0.30	(0.27,0.33)	0.30	(0.28,0.33)
High school or more	0.23	(0.22,0.24)	0.15	(0.13,0.18)	0.21	(0.18,0.25)	0.28	(0.25,0.31)	0.25	(0.23,0.27)
Rural locality (2500 or less inhabitants)	0.21	(0.20,0.23)	0.26	(0.23,0.29)	0.26	(0.23,0.30)	0.17	(0.15,0.19)	0.19	(0.17,0.21)**
Year**										
1992	0.12	(0.10,0.15)	0.13	(0.10,0.16)	0.23	(0.19,0.28)	0.09	(0.07,0.12)	0.11	(0.08,0.13)
1997	0.15	(0.13,0.16)	0.21	(0.18,0.23)	0.18	(0.15,0.21)	0.13	(0.11,0.16)	0.11	(0.10,0.13)
2006	0.17	(0.16,0.19)	0.15	(0.12,0.18)	0.14	(0.11,0.18)	0.16	(0.14,0.19)	0.20	(0.18,0.23)
2009	0.24	(0.23,0.26)	0.20	(0.18,0.23)	0.24	(0.21,0.27)	0.28	(0.26,0.31)	0.25	(0.23,0.27)
2014	0.31	(0.30,0.33)	0.32	(0.29,0.35)	0.21	(0.18,0.24)	0.33	(0.31,0.36)	0.34	(0.31,0.36)

Among those who report ever using a method. Survey weights were used to account the complex survey design. Sterilized women were excluded (n = 79)

* *p* < 0.05; ** *p* < 0.001

^a^Continuous variable (number of years of schooling a woman would be expected to have given her age minus number of years of schooling reported by each woman)

^b^Women could mention more than one contraceptive method, proportion more than 1

In all our multivariate logistic regression models, LARC use is the comparison group; odds ratios (OR) greater than one in each model indicate higher odds of LARC use compared to tier 2, tier 3, or non-use. Women who reported knowledge of tier 2 methods (as the most effective method known) have lower odds of LARC use compared to women with knowledge of tier 1. The association was significant compared with use of tier 2 methods (OR = 0.182; CI 95 % 0.126–0.263; Table 2a), use of tier 3 methods (OR = 0.190; CI 95 % 0.129–0.278; Table 2b) and non use (OR = 0.210; CI 95 % 0.148–0.299; Table 2c).

**Table 2:** Association between socio-demographic characteristics and current LARC use versus tier 2 or 3 or no method, among female adolescents (15–19), Mexico ENADID 1992–2014

	Panel A		Panel B		Panel C	
	Tier 2: Hormonal (pills/injection/patch) versus Tier 1 (LARC)		Tier 3 (Condom/sponge/traditional) versus tier 1 (LARC)		None versus tier 1 (LARC)	
	n = 3989 (N = 1,284,614)		n = 4669 (N = 1,620,405)		n = 6381 (N = 2,186,517)	
	OR	CI (95 %)	OR	CI (95 %)	OR	CI (95 %)
Contraceptive Knowledge (Ref:Tier 1)^b^						
Tier 2 (Hormonal)	0.182**	(0.126–0.263)	0.190**	(0.129–0.278)	0.210**	(0.148–0.299)
Tier 3 (Condom, sponge, traditional)	0.805	(0.272–2.384)	0.082**	(0.039–0.169)	0.269**	(0.136–0.532)
None	0.841	(0.482–1.468)	0.262**	(0.204–0.641)	0.816	(0.492–1.353)
Individual characteristics						
Age (Ref:15–16)	0.969	(0.882–1.066)	1.085	(0.994–1.184)	1.084*	(1.009–1.164)
Educational gap^a^	1.061*	(1.002–1.124)	1.103**	(1.034–1.176)	1.080*	(1.015–1.148)
Married or co-habiting exposure	1.643**	(1.180–2.289)	6.333**	(4.825–8.314)	6.703**	(5.237–8.579)
Currently working	0.667**	(0.510–0.873)	1.106	(0.867–1.411)	0.935	(0.743–1.177)
Household characteristics						
Man as head of household	1.06	(0.813–1.383)	1.026	(0.807–1.304)	1.159	(0.929–1.447)
Head of household schooling (Ref: None)						
Primary	1.027	(0.673–1.566)	0.912	(0.571–1.457)	1.276	(0.854–1.906)
Secondary	1.021	(0.644–1.618)	0.796	(0.485–1.307)	1.145	(0.748–1.753)
Highschool	0.638	(0.400–1.018)	0.507**	(0.310–0.830)	0.873	(0.569–1.341)
Rural locality (2500 or less inhabitants)	0.825	(0.655–1.041)	1.052	(0.837–1.322)	1.002	(0.826–1.217)
Year (Ref: 1992)						
1997	2.178**	(1.520–3.122)	1.104	(0.748–1.630)	2.083**	(1.471–2.948)
2006	2.078**	(1.354–3.189)	0.944	(0.608–1.465)	1.261	(0.849–1.873)
2009	1.796**	(1.267–2.547)	0.863	(0.590–1.261)	1.331	(0.952–1.860)
2014	3.067**	(2.178–4.318)	1.119	(0.785–1.593)	1.665**	(1.215–2.283)

Among those who report ever using a method. Models adjusted by region based on grouping the 32 Mexican States by average socioeconomic level

* *p* < 0.05; ** *p* < 0.001

^a^Continuous variable (number of years of schooling a woman would be expected to have given her age minus number of years of schooling reported by each woman)

^b^We classified each woman into the highest tier method she reported knowing

We found the same pattern (lower odds of LARC methods use) when we compared knowledge of tier 3 methods (as the most effective method known) with knowledge of tier 1 methods. The association was significant compared with use of tier 3 methods (OR = 0.082; CI 95 % 0.039–0.169; Table 2b) and with non use (OR = 0.269; CI 95 % 0.136–0.532; Table 2c). No knowledge was negatively associated with LARC use compared with knowledge of tier 1 methods, the relation only was significant compared with use of tier 3 methods (OR = 0.262; CI 95 % 0.204–0.641; Table 2b).

Associations were noted between key sociodemographic variables and LARC use. Exposure to marriage was positively associated with current use of a tier 1 methods compared with tier 2 (Table 2a, OR = 1.64; 95 % CI 1.80–2.28), tier 3 (Table 2b, OR = 6.33; 95 % CI 4.82–8.31), and non use (Table 2c, OR = 6.70; 95 % CI 5.23–8.57). Each additional year of educational gap was positively associated with LARC use; women with larger educational gaps had higher odds of LARC use (Table 2a–c). Survey year was also significantly associated with LARC use. Compared with 1992, women in 1997, 2006, 2009 and 2014 waves had significantly higher odds of LARC use compared to tier 2 methods use (Table 2a).

In the subsample of women who reported a pregnancy (n = 6913; Table 3), the associations between use of tier 1 methods and knowledge of contraceptive tiers is similar as in the full sample. However, marriage was no longer significantly associated with LARC use compared to use of tier 2 or tier 3 methods.

**Table 3:** Association between socio-demographic characteristics and current LARC use versus tier 2 or 3 or no method, among ever pregnant female adolescents (15–19), Mexico ENADID 1992–2014

	Ever pregnant					
	Panel A		Panel B		Panel C	
	Tier 2: Hormonal (Pills/injection/patch) versus tier 1 (LARC)		Tier 3 (Condom/sponge/traditional) versus tier 1 (LARC)		None versus tier 1 (LARC)	
	n = 3395 (N = 1,145,699)		n = 3161 (N = 1,118,503)		n = 4370 (N = 1,555,101)	
	OR	CI (95 %)	OR	CI (95 %)	OR	CI (95 %)
Contraceptive Knowledge (Ref:Tier 1)^b^						
Tier 2 (Hormonal)	0.213**	(0.146–0.311)	0.254**	(0.168–0.384)	0.261**	(0.182–0.374)
Tier 3 (Condom, sponge, traditional)	0.544	(0.201–1.468)	0.109**	(0.050–0.236)	0.351*	(0.159–0.775)
None	1.236	(0.637–2.398)	0.505*	(0.283–0.898)	1.253	(0.726–2.160)
Individual characteristics						
Age (Ref:15–16)	0.899	(0.808–1.000)	0.984	(0.879–1.101)	1.029	(0.951–1.115)
Educational gap^a^	1.022	(0.963–1.084)	1.013	(0.947–1.084)	1.031	(0.971–1.096)
Married or co-habiting exposure	0.676	(0.427–1.071)	0.893	(0.579–1.377)	2.692**	(2.047–3.540)
Currently working	0.676*	(0.495–0.923)	1.141	(0.846–1.540)	0.967	(0.755–1.239)
Household characteristics						
Man as head of household	1.056	(0.782–1.426)	0.887	(0.654–1.205)	1.155	(0.907–1.470)
Head of household schooling (Ref: None)						
Primary	1.019	(0.665–1.561)	0.981	(0.591–1.627)	1.151	(0.768–1.724)
Secondary	1.01	(0.629–1.622)	0.789	(0.461–1.351)	1.039	(0.676–1.597)
Highschool	0.822	(0.503–1.345)	0.576	(0.329–1.009)	0.877	(0.564–1.365)
Rural locality (2500 or less inhabitants)	0.773*	(0.606–0.987)	1.036	(0.807–1.331)	0.986	(0.802–1.212)
Year (Ref: 1992)						
1997	2.277**	(1.553–3.338)	1.006	(0.663–1.528)	2.084**	(1.448–3.000)
2006	2.065**	(1.317–3.236)	0.89	(0.555–1.426)	1.003	(0.665–1.513)
2009	2.100**	(1.457–3.026)	0.8	(0.524–1.221)	1.146	(0.809–1.623)
2014	3.290**	(2.302–4.701)	1.371	(0.920–2.045)	1.525*	(1.094–2.126)

Among those who report ever using a method. Models adjusted by region based on grouping the 32 Mexican States by average socioeconomic level

* *p* < 0.05; ** *p* < 0.001

^a^Continuous variable (number of years of schooling a woman would be expected to have given her age minus number of years of schooling reported by each woman)

^b^We classified each woman into the highest tier method she reported knowing

## Discussion

Our study demonstrates an important disparity in use of the most effective forms of contraception by adolescents. Among adolescents who have had a pregnancy, LARC use has increased from 24 % in 1992 to 37 % in 2014. However, among the subset of adolescents who have never been pregnant, current LARC use has remained unacceptably low: 1 % in 1992 and 2 % in 2014. This difference represents an important missed opportunity to prevent primary adolescent pregnancy in Mexico. Our results show that use of LARC is strongly correlated with exposure to marriage, but our data also suggest that it is previous pregnancy that may be the true driver of LARC use. Although marriage and pregnancy are highly correlated, in a subsample of women who had experienced a previous pregnancy, marriage was no longer correlated with LARC use compared to tier 2 or 3 methods use. However, marriage remains highly correlated with LARC use compared to not using a method.

Our results suggest that an important step in increasing use of LARC by adolescents may be improved contraceptive knowledge. We found a significant, positive association between LARC use and knowledge of tier 1 methods. However, we do not know whether adolescents know about LARC after or before uptake of the method. There is no clear evidence about the causal relationship between knowledge of contraceptive methods and use of methods among adolescents, but it is agreed that knowledge is necessary, if not sufficient, for improving use [19]. Low contraceptive knowledge and limited access remain key barriers to using contraceptives for adolescents [9, 19]. Efforts to improve knowledge among adolescents include school-based health centers as an alternative access to methods and knowledge [5], school based interventions such as sex education programs that include inside and outside classroom activities [3, 4, 29] and peer educator models, youth friendly services, health facility-based activities and community-based sex education programs [35]. While some studies of these interventions demonstrated a small increase in the use of contraception [4], the majority of the interventions did not report a significant change in method use [3, 29, 35]. Strategies beyond those focusing on strengthening adolescent knowledge and access are needed to improve adolescent contraceptive use.

Perceptions about the risk of pregnancy may also play a role in use of contraceptive methods. Adolescents in Mexico may not perceive themselves to be at risk of pregnancy until a pregnancy happens, which could explain our findings about LARC use and previous pregnancy. Provider misconceptions about LARC safety in adolescents, or judgment regarding sexual activity among unmarried adolescents may also contribute to our findings. It is not uncommon for women to enter healthcare via antenatal and delivery care, our previous work suggests that much LARC uptake is in the immediate post-partum setting prior to discharge from place of delivery [14]. Educating both adolescents and providers about the safety, efficacy and availability of LARC use is an important strategy to reduce unintended pregnancy.

LARC use by adolescents is higher in Mexico than in the United States (US), where only 5 % of contraceptive users choose a LARC method [18]. Data on adolescent LARC use in other parts of the world are very scarce and limited to specific populations, such as married adolescents [17]. In Mexico, despite relatively high use of IUDs by adolescents who report ever using a method, as our results show, overall use in the adolescent population remains much lower than by older women [2]. Evidence in Mexico about the proportion of LARCs placed in the immediate post-partum setting versus interval placements in primary care or other settings are extremely limited. National family planning policy in Mexico states that LARC methods should be available to adolescents and they should have access to them across all types of insurers (i.e. *Seguro Popular* and employment-based insurance) and facilities [11]. Little is known about the reality of LARC provision outside of hospital settings, where uptake is concentrated [14]. Immediate post-partum LARC placement is useful for reducing rapid repeat pregnancy but does not contribute to preventing unwanted first pregnancies among adolescents.

This study must be interpreted with the following limitations in mind. First, use of contraceptive methods was by self-report, which may be subject to recall bias. Second, we have no information on reasons for sterilization. Third, we have no information about temporality (e.g. of knowledge and ever or current use) or continuation. Fourth, we have no information about sexual activity or women’s perception of risk of pregnancy; to address this limitation we conducted an additional analysis among the subsample of adolescents who reported ever experiencing a pregnancy. Despite these limitations, our study makes important contributions to knowledge about LARC use in adolescents. An important aspect of our study is the population-based sample (of adolescents who report knowledge and ever use of a method); many previous studies of contraceptive use in adolescents included only married adolescents. In countries such as Mexico, marriage is no longer an accurate proxy for sexual activity [2].

Despite international and national guidelines and demonstrated safety and efficacy of LARC in adolescents, including nulliparous adolescents [10, 15, 32, 36], studies continue to document both demand and supply-side barriers to use of LARC in adolescents [6, 28, 31]. Mexico can serve as a model for other countries seeking to increase LARC use by adolescents. LARC use is relatively high among female adolescents who are currently using modern methods, but remains primarily tied to having had a pregnancy. Our study highlights the need to expand access to LARC methods beyond married women or the post-partum setting, including education and practical training for health care providers in primary care [30].
